# Laser treatment of oral vascular anomalies. A retrospective observational study

**DOI:** 10.4317/medoral.27681

**Published:** 2025-10-17

**Authors:** Luis Monteiro, Carla Fazendeiro, Sara Ferreira, Leonor Delgado, José Júlio Pacheco, Filomena Salazar

**Affiliations:** 1 UNIPRO, Oral Pathology and Rehabilitation Research Unit, University Institute of Health Sciences (IUCS-CESPU), Gandra 4585-116, Portugal; 2 Medicine and Oral Surgery Department, University Institute of Health Sciences (IUCS-CESPU), Gandra 4585-116, Portugal; 3 Animal and Veterinary Sciences Department, University Institute of Health Sciences (IUCS-CESPU), Advanced Polytechnic and University Cooperative, CESPU CRL, 1317, 4585-116 Gandra, Portugal; 4 GIPOC-Comparative Oral Pathology Research Group, University Institute of Health Sciences-Advanced Polytechnic and University Cooperative (IUCS-CESPU), 4585-116 Gandra, Portugal

## Abstract

**Background:**

Oral vascular anomalies, though benign, may impact comfort and aesthetics, particularly in visible or functional areas. Laser therapies have gained prominence due to their precision, minimal invasiveness, and favourable outcomes. This study aimed to analyse the laser treatment modalities for oral vascular anomalies and their respective outcomes in a population of the north of Portugal.

**Material and Methods:**

This retrospective study analysed 111 vascular anomalies in 95 patients treated between January 2011 and May 2025 in the University Clinics of IUCS-CESPU. Data on demographics, type and location of vascular anomalies, treatment (Nd:YAG, CO2 or diode laser), and outcomes were collected.

**Results:**

Venous lakes were the most frequent diagnosis (n=77;69.4%) and lip was the most common location (n=81;73%). Induced photocoagulation (IPC) (n=79;71.2%), essentially with Nd:YAG, was the predominant technique. Recurrence was observed in 4 cases (3.6%), healing occurred mostly 21 days (n=88; 79.3%), scar tissue was present in 10 cases (9%), pain was reported in 29 patients (26.1%) (mean of 0.85±0.14), and satisfaction was high (n=103; 92.8%). No major complications were observed. Logistic regression analysis showed that larger vascular anomalies were associated with delayed healing (p=0.009) and scar formation correlated with presence of pain (one day after intervention) (p=0.03) and recurrence (p=0.039).

**Conclusions:**

Laser-based management in particular, IPC, is a safe, effective, and well tolerated option for the treatment of small oral vascular anomalies. Prospective studies are encouraged to optimize protocols and standardize the technique.

## Introduction

Vascular anomalies represent a heterogeneous group of blood vessel disorders, characterized by variable frequency, appearance, and clinical impact. They result from dysfunctions of the endothelium and associated tissues, leading to abnormal vascular growth. These anomalies may be congenital or acquired, and can occur in various anatomical regions, with a marked predilection for the head and neck (1 - 4).

These vascular anomalies encompass a broad spectrum of clinical presentations, historically with heterogeneous definitions (5), but are now classified, according to the International Society for the Study of Vascular Anomalies (ISSVA), into two main categories: vascular tumours and vascular malformations (6 , 7). Among the most common acquired forms are varices and venous lakes, which are more frequently observed on the lip in elderly patients (8 , 9).

Vascular malformations are categorized according to the type of vessels involved: capillary, venous, lymphatic, or arteriovenous. Some forms combine multiple vascular components. Unlike vascular tumours, which typically follow a proliferative phase followed by involution, vascular malformations do not regress spontaneously. They tend to persist or slowly enlarge over time, particularly when triggered by factors such as trauma, hormonal fluctuations, or infections (2 , 4 - 7).

Although most vascular anomalies are benign, certain forms may lead to more severe complications, including haemorrhages, ulcerations, infections, or tissue destruction, especially during dental or surgical procedures. They may also cause aesthetic concerns or functional impairments (1 , 10 - 13). These are the main reasons for treating vascular anomalies.

In the absence of histological confirmation, diagnosis often relies on clinical examination, and imagiological exams (including ultrasound, magnetic resonance imaging, and/or angiography). However, the clinical diagnosis of vascular anomalies can be particularly challenging due to their variable presentation in the oral cavity and the potential overlap with more common mucosal lesions. Moreover, their low prevalence in dental settings may lead to under recognition or delayed referral, increasing the risk of complications during invasive procedures (1 , 3 , 4 , 12 , 14).

Various therapeutic approaches have been proposed for managing oral vascular anomalies, including surgical excision, sclerotherapy, electrosurgery, and cryotherapy (3 , 4 , 9 , 12 , 14 - 17). However, laser therapy is a mainstay of management of mucosal and skin vascular malformations using various wavelengths, irradiation parameters, and application techniques (9 , 12 , 13 , 16). It has generated growing interest due to its minimally invasive nature, reduced intra and postoperative bleeding, improved patient comfort, and generally favourable aesthetic outcomes. Several types of lasers, such as Nd:YAG, diode, and CO2 lasers, are currently used in clinical practice, each with specific physical properties and indications, allowing for photocoagulation, vaporization, or tissue excision. They have been found to be safe and effective in the treatment of anomalies, particularly using induced photocoagulation (IPC). This is a laser-based technique that involves the gradual thermal alteration of vascular endothelial cells, leading to progressive vessel closure over time. Unlike ablative methods, IPC relies on sub-threshold energy levels to induce coagulation without immediate tissue removal, making it particularly suitable for lesions located in functionally or aesthetically sensitive areas. However no specimen would be available for histopathologic examination (9 , 12 , 13 , 16 , 18 - 20).

Despite the increasing use of laser therapy, scientific data on its application in the treatment of oral vascular anomalies remain heterogeneous and fragmented. Most available publications consist of case reports, small clinical series, or technical descriptions, providing little comparative data. Furthermore, there is currently no widely accepted clinical protocol (specially referring to laser´s settings) or standard of care for the diagnosis and management of these lesions in the field of dentistry, which further highlights the need for more comprehensive studies. As a result, clinicians still have limited standardized recommendations regarding the most appropriate treatment strategy, particularly in dental settings (19 , 20).

In this context, gaining deeper knowledge on vascular anomalies, particularly in terms of clinical presentation, diagnostic methods, and therapeutic options, is essential to improve patient safety and treatment efficacy. It is with this in mind that the main objective of this study is to retrospectively evaluate the therapeutic modalities and outcomes of oral vascular anomalies with a particular focus on the results and safety of laser-based treatments.

## Material and Methods

Study characterization

This retrospective observational study investigates a cohort of cases of vascular anomalies who underwent laser treatment at the University Clinics of IUCS - CESPU. The study received the approval from the Institutional Ethics Committee of the University Institute of Health Sciences (project Laseroral_CESPU_2018; 01/CE-IUCS/2024). All aspects of the research were carried out in accordance with the ethical principles outlined in the 1964 Helsinki Declaration and its subsequent revisions, or equivalent ethical guidelines. The data used in this analysis were obtained from anonymized clinical records, which were thoroughly coded to safeguard patient confidentiality and privacy. Additionally, informed consent was secured from all patients before their respective interventions.

Sample constitution

The sample was compiled using a non-probabilistic convenience sampling approach. Cases were gathered from clinical records at the university clinic and from the Laser Unit and Oral Medicine Postgraduation program at IUCS-CESPU. Data collection spanned from January 2011 to May 2025.

Inclusion criteria

Patients included in this study attended the university clinic, the Oral Laser Unit, and the Oral Medicine Postgraduation program at IUCS-CESPU during the data collection period. Eligible participants presented with one or more clinically confirmed oral vascular anomalies that had no prior treatment and were subsequently managed with oral laser treatment. All included cases underwent a thorough medical and clinical examination. When indicated (e.g., for lesions larger than 2 cm), imaging examinations such as eco-doppler, magnetic resonance imaging, or angiography were performed. Only cases with a clear indication for treatment, including aesthetic concerns, functional impairment, or the need for histological evaluation (in cases of excision procedures), were considered eligible for intervention.

Exclusion criteria

Some patients were excluded from the study as result of: (1) Lesions lacking treatment indications related with their anatomic/physiologic constitution (e.g., pulsatile vascular lesions, cases with an imaging diagnosis of arteriovenous malformations, predominantly arterial composition, high-flow rates, or an indefinable nature); (2) cases extending beyond the oral location or associated with systemic anomalies from syndromic-associated lesions (e.g., Sturge-Weber disease); (3) vascular tumours (e.g., infantile haemangiomas); (4) cases where patients had undergone therapy with immunosuppressants or other forms of systemic immunosuppression; and (5) cases with insufficient clinical and follow-up information (less than 2 months).

From an initial pool of 138 potential cases diagnosed with vascular anomalies, 27 were excluded. Of these, 12 did not undergo laser treatment (8 were arteriovenous malformations or high-flow lesions, 2 had a predominant arterial constitution, and 2 extended beyond the oral cavity). The remaining 15 excluded cases, despite having received laser treatment, lacked the necessary information for the study, primarily comprehensive follow-up assessments. This selection process resulted in a final sample of 95 patients and 111 lesions, comprising 49 males and 46 females, with ages ranging from 8 to 84 years.

Data collection

Data were systematically gathered from existing patient clinical records. Each included case underwent a comprehensive medical history interview and a thorough clinical examination of the oral cavity, performed with a sterile kit to enable detailed observation of oral manifestations, and including a diascope manuever with a slide glass (Thermo Scientific Nunc Microscope Slides, Waltham, USA). When clinically indicated-for instance, for pulsatile lesions or those exceeding 2cm in diameter-supplementary imaging studies were performed. These modalities included ultrasonography with Eco-Doppler, magnetic resonance imaging (MRI), or angiography. In the case of antitrombotic/anticoagulant medication we followed the indication of general medical practitioner (GP), usually with indication for stopping the medication before the procedures according to GP guidelines.

All pertinent information extracted was subsequently compiled into an anonymized and coded Microsoft Excel spreadsheet (Microsoft Corporation, 2024, Redmond, WA). The variables recorded encompassed: patient age, gender, definitive diagnosis (categorized according to the International Society for the Study of Vascular Anomalies classification (6), the specific anatomical location of the lesion (coded using ICD-10: D10.0-10.3), the lesion's maximum clinical diameter in centimetres, the chosen intervention type and the specific laser employed, any intraoperative complications encountered, the duration until complete cicatrisation, postoperative complications, reported pain levels, the presence of scarring, patient satisfaction scores, and any instances of recurrence.

Interventions

Prior to laser irradiation, a 2% lidocaine with 1:200,000 epinephrine solution was injected perilesionally. Patients were assigned to intervention or laser type groups without specific randomization, except when a histological report was required, in which case an excision procedure was chosen. All procedures were performed by a single operator (LM), adhering to standard safety precautions for the operator, patient, and assistant.

We categorized the interventions into two types: "excision" and "induced photocoagulation (IPC)". The lasers used were categorized by their three distinct wavelengths: CO2 (10,600nm), Nd:YAG (1064nm), and diode (980nm). As a non-probabilistic convenience sampling, the selection of lasers was guided by their availability within our laser unit and the specific indication of their wavelengths for treating vascular anomalies. For example, the Nd:YAG (1064nm) and diode (980nm) lasers were chosen due to haemoglobin being their target chromophore, excellent for IPC, while the CO2 (10600nm) laser (with water as cromophore) was selected for its high coagulation capacity, used mainly for excision.

The excision procedure, performed with either the CO2 (10,600nm) or Nd:YAG (1064nm) laser, involved the cutting and removal of the lesion with a 2-3mm margin of healthy tissue. The obtained specimen was submitted for routine histological examination, with specific notation of laser excision. The resulting wound was allowed to heal by secondary intention.

The IPC procedure utilized either diode (980nm) or Nd:YAG (1064nm) lasers. This involved transmucosal irradiation of the lesion with a non-activated fiber, held approximately 2mm from the lesion's surface. A continuous inward rotatory movement was employed, starting from the periphery and moving towards the center, ensuring the fiber never remained static. This was continued until a slight superficial whitening or reduction in lesion size was observed. When using an R30 (FOTONA®, Slovenia) handpiece, non-superimposing circular spots were applied using the same technique (if spot didn't covered the entire lesion) until a colour change or slight contraction of the lesion were noted. Any potential tissue wound was permitted to heal by secondary intention.

Specific parameters were utilized for each laser type. The CO2 laser (DEKA Smart US20D, Firenze, Italy) operated in pulse mode (50 Hz) with an output power of 4.5W. It featured a 1mm spot size and a 0.5mm focal distance, yielding a theoretical power density of 573.25 W/cm² and a fluence of 11.46 J/cm². The beam was focused for mucosal cutting using an angulated mirror handpiece.

For Nd:YAG laser (1064nm wavelength - FOTONA®, Lightwalker ATS, Slovenia) excision, parameters included an output power of 3.5W and a frequency of 70Hz (theoretical power density: 4375 W/cm²; fluence: 62.5 J/cm²), used in short pulse (SP) mode with a 320-µm fiber. For IPC using the Nd:YAG laser, the parameters were an output power of 5W, 70 Hz, SP mode, defocused at 2mm with a 320-µm fiber (theoretical power density of 1086.96 W/cm²; fluence: 8.15 J/cm²). When employing the R30 (FOTONA®, Slovenia) handpiece, a 4mm spot, 1.5Hz pulse repetition, 25ms pulse duration, and 75 J/cm² fluence were used.

The diode laser (980nm - Lasotronix Smart M, Piaseczno, Poland) parameters involved a non-contact mode with a 2mm focal distance, utilizing a 200-µm fiber. The laser operated in continuous mode with 3W power, delivering a fluence/power density of 20 J/cm².

Patients received post-intervention instructions to abstain from consuming hard and hot foods for the initial 2-3 days. Analgesic or anti-inflammatory medications were recommended only when needed by the patients as experiencing pain, discomfort, or other complications.

Evaluation after intervention

Patients underwent weekly follow-up examinations for the first month post-intervention, followed by annual assessments, with the date of the last follow-up appointment recorded. Photographs were taken at each stage of the procedure and during subsequent assessments.

Several variables were collected, including the occurrence of intraoperative complications (e.g., hemorrhage, pain, discomfort) and the time to cicatrization (measured in days within a weekly period). Postoperative complications (e.g., paresthesia, hemorrhage, pain, scarring) were also documented. Specifically, pain was evaluated using a Visual Analogue Scale (VAS) at one day, three days, seven days, and beyond seven days post-intervention. We further assessed the presence of scarring using a dedicated scar scoring system (Supplementary 1 - http://www.medicina.oral.com/carpeta/suppl1_27681), a patient satisfaction score (ranging from 1 for "not satisfied" to 5 for "very satisfied"), and the presence and date of any recurrence (recorded in months).

Statistical analysis

Data analysis was performed using IBM SPSS Statistics (version 30). Quantitative variables, such as age and lesion size, were described using mean and standard deviation. Categorical variables, including sex, lesion location, lesion type, treatment type, and clinical outcomes were expressed as frequencies and percentages. Comparisons between categorical variables were performed using the Chi-square test or Fisher's exact test when expected frequencies were below 5. The significant results were included in a logistic binary regression model to determine the independent value of the variables in a multivariate analysis.

To evaluate the recurrence-free interval time, Kaplan-Meier survival univariate analysis was conducted, and the log-rank test was used to assess differences in recurrence-free interval time between different variables. Cox regression model was used to perform a multivariate analysis of the variables with significant effect in univariate analysis.

The threshold for statistical significance was set at p&lt;0.05.

## Results

Sample characterization

The study included 95 patients with an average age of 54.93±1.74 years, ranging from 8 to 84 years old. The cohort comprised 49 males (51.6%) and 46 females (48.4%). A total of 111 lesions from these 95 patients were analysed. Regarding the gender distribution of the lesions, 61 lesions (55%) were observed in male patients, and 50 lesions (45%) were found in female patients.

Most patients (n=68;71.6%) reported no significant systemic diseases or comorbidities. However, some patients presented with the following conditions: arterial hypertension and other cardiovascular disease (n=15;15.8%), diabetes mellitus (n=6;6.3%), history of stroke (n=1;1.1%), depression (n=1;1.1%), kidney disease (n=1;1.1%). Other reported conditions, each affecting one patient (1%), included thyroid disorders, oral and cutaneous lichen planus, and a history of lip cancer.

Distribution of Oral Vascular Anomalies

Venous lake was the most frequent clinical diagnosis, accounting for 77 lesions (69.4%). Vascular malformations were the second most common, observed in 11 cases (9.9%), followed by pyogenic granuloma (10 cases, 9%), vascular anomalies (not otherwise specified) (9 cases, 8.1%), spider angiomas (2 cases, 1.8%), angiofibroma (1 case, 0.9%), and ruby angioma (1 case, 0.9%) (Table 1).

Table 1

Of the 111 clinically diagnosed lesions, 25 (22.5%) underwent histopathological examination. Most of these histological diagnoses were hemangiomas (n=13; 52%), followed by pyogenic granuloma (n=10, 40%), and one case each of a vascular malformation (4%) and an angiofibroma (4%).

The most frequent location for the lesions was the lips, accounting for 81 cases (73%), with the majority found on the lower lip (n=56; 69.1% of lip lesions). Other locations included the buccal mucosa in 12 cases (10.8%), the tongue in 9 cases (8.1%), and the vestibular mucosa in 5 cases (4.5%), all situated at the maxillary level.

Regarding lateralization, where data was available, 41 lesions (41%) were located on the right side, 38 (38%) on the left side, and 21 (21%) were positioned at the midline.

The clinical size of the lesions varied from 0.2 cm to 4 cm, with a mean size of 0.7±0.048 cm. Fifty-seven lesions (51.4%) measured 0.6 cm or more, a cutoff chosen to represent the 50th percentile of the variable for statistical analysis.

Intervention and Outcomes

Type of Treatment

**Figure 1:**
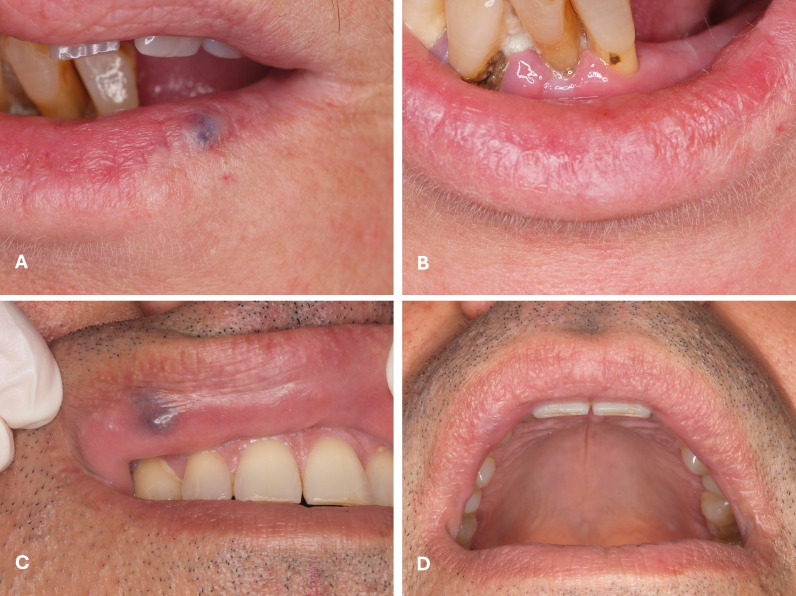
A) Excision of a left lower lip venous lake in a 66-year-old female patient with CO2 laser 10,600nm and respective 12 months follow-up image (B); C) Excision of right upper lip venous lake in a 53-year-old male patient with CO2 laser 10,600nm and respective 12-months follow-up image (D).

**Figure 2:**
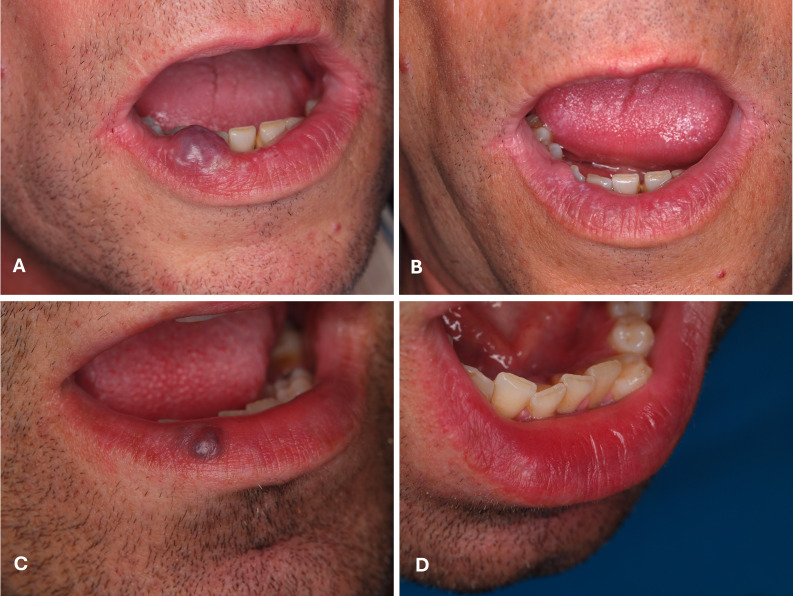
A) vascular malformation in the right lower lip in a 45-years-old male patient submitted to induced photocoagulation with diode 980 nm laser; B) respective control image after 12 months of follow-up; C) a venous lake in the right lower lip in a 42-years-old male patient submitted to induced photocoagulation with diode 980 nm laser and respective control image after one month of follow-up (D).

**Figure 3:**
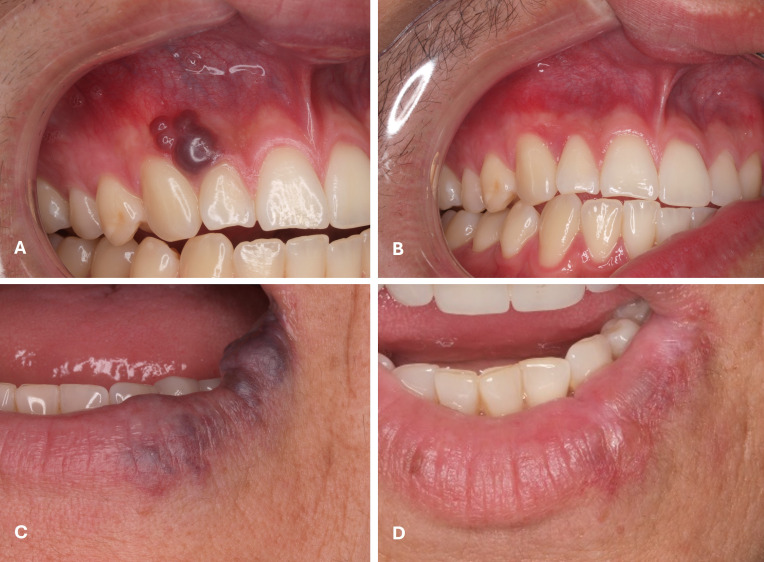
A) vascular malformation in right maxillary vestibular oral mucosae of an 18-years-old male patient submitted to induced photocoagulation with Nd:YAG 1064nm laser using a 4-mm spot diameter (R30 handpiece) and respective follow-up image of 12 months of follow-up (B); (C) a left lower lip vascular malformation in a 59-years-old female patient submitted to induced photocoagulation with a Nd:YAG 1064 nm laser using a 320µm fiber and respective control image after 4 years of follow-up (D).

The type of treatment corresponded to either excision (n=32; 28.8%) or induced photocoagulation (IPC) (n=79; 71.2%). The procedures were performed using three types of lasers: Nd:YAG laser in 78 cases (70.3%), CO2 laser in 22 cases (19.8%), and diode laser in 11 cases (9.9%) (Table 1) (Figures 1 to 3).

1

2

3

Regarding the handpiece used, most IPC procedures were performed with the fiber tip (n=55), while 24 were conducted using the R30 handpiece. Overall, among all cases, 65 treatments (58.6%) involved the fiber handpiece, 24 (21.6%) the R30 handpiece, and 22 (19.8%) the mirror handpiece.

There were no complications during surgery (including haemorrhage, pain, etc). No need for suture was reported.

Postoperative Follow-Up

Several variables were assessed at one, two, and three weeks, and at one month of follow-up. Recurrence status was also evaluated at the last follow-up appointment. The mean follow-up duration for the included patients was 19.07±2.3 months, with a range of 2.03 to 103.33 months. Over this period, recurrence was observed in 4 cases (3.6%).

Most cases (88; 79.3%) achieved complete cicatrisation within 21 days. Two cases (1.8%) required a longer healing period, while 23 cases (20.7%) healed in less than 21 days.

Regarding scarring, 10 cases (9%) presented some grade of scar tissue (visible in only 6 cases; 5.4%), whereas 101 cases (91%) showed no scar. According to scar scoring, 101 cases (91%) scored 0, four cases (3.6%) scored 1, four cases (3.6%) scored 2, and two cases (1.8%) received a score of 3 (Table 1).

Postoperative pain was evaluated using the Visual Analog Scale (VAS) at three time points: one day, three days, and seven days after surgery. On the first day after intervention, pain of any grade was reported in 29 patients (26.1%) (mean of 0.85±0.14), with the most frequent scores being VAS "3" (9.9%) and VAS "4" (5.4%). After three days, pain was still present in 20 cases (18%) (mean of 0.43±0.95), with the most frequent score being VAS "2" and "3"(7.2 each%). After 7 days only 3 cases (2.7%) (mean of 0.03±0.015) referred mild/discomfort pain (both scoring "1" from 0 to 10 VAS scale) (Table 1).

Medication was prescribed postoperatively in 23 cases (20.7%), while 88 patients (79.3%) did not require any pharmacological support.

Finally, patient satisfaction was evaluated using a five-point scale (from 1: Very dissatisfied, to 5: Very satisfied) (Table 1). Most of them were 4 (n=58;52.3%), or 5 (n=45;40.5%).

Comparative Analysis

To further investigate factors influencing clinical outcomes in patients treated for oral vascular anomalies, a comparative analysis was conducted based on four key endpoints: postoperative pain, cicatrisation time, presence of scarring, and lesion recurrence. Recurrence was also analysed by recurrence-free interval time using Kaplan-Meier curves. Variables selected for comparison included demographic characteristics, lesion features, and treatment-related factors.

Pain (after one day of intervention) and Cicatrisation Time

We found significant associations between postoperative pain and lesion size (Supplementary 1 - http://www.medicina.oral.com/carpeta/suppl1_27681). Specifically, larger lesions (&gt;0.6 cm) were associated with more pain (p=0.002). Surgical technique was also significantly associated where excision and CO2 laser type resulted in more pain compared to induced photocoagulation (IPC) and other lasers (p&lt;0.001 and p=0.003, respectively). Patients whose healing time was under 21 days were significantly less likely to report pain (p=0.042). Scar formation was significantly more common in cases with presence of pain (p=0.011).

Analysing time of healing we observed a significant association with size (p=0.003), type pf laser (p=0.028), presence of pain (one day after) (p=0.042). This Implies that bigger lesions, treated with CO2 laser or with pain (one day after) were associated to more healing time (&gt;21days).

Among Nd:YAG laser handpieces, the fiber HP was associated with a higher cicatrisation time compared to the R30 HP (p=0.031).

We also evaluate the relation of these variables using a logistic regression specially to determinate their independent effect. For the presence of pain (after one day), none of them reveal significance in the multivariate analysis. On the cicatrization time, the size of the lesion (p=0.021, Exp of 3.72, 95% IC 1.22-11.52) was the only independent and significant association (Supplementary 1: http://www.medicina.oral.com/carpeta/suppl1_27681). In a parallel analysis for the cases using Nd:YAG laser, the type of handpiece lost significant in the model including the other significant variables in univariate analysis (p=0.067, Exp of 0.35, 95% IC 0.18-1.07).

Scar Presence and Recurrence

The only variables with a significant association with the presence of scar was the presence of pain (one day after) (p=0.011) and the presence of recurrence (p=0.04) (Supplementary 1: http://www.medicina.oral.com/carpeta/suppl1_27681). Focusing recurrence, we found a significant association with lesion location (p=0.011). Recurrences were more evident in the vestibular oral mucosa and gums, though this observation might be influenced by the smaller number of cases in these two locations.

On multivariate analysis using a logistic binary regression of the presence of scar, we observed an independent and significant effect of the presence of pain (one day after) (p=0.030; Exp of 4.71, 95% IC 1.16-19.03) and with recurrence (p=0.039; Exp of 10.48, 95% IC 1.12-97.95). Focusing recurrence on the other hand, this was also true for presence of scar (p=0.012; Exp of 2.44, 95% IC 1.21-4.89) and location (p=0.024; Exp of 2.25, 95% IC 1.13-4.56) (Supplementary 1: http://www.medicina.oral.com/carpeta/suppl1_27681).

Recurrence-Free Time (Kaplan-Meier Analysis)

**Figure 4:**
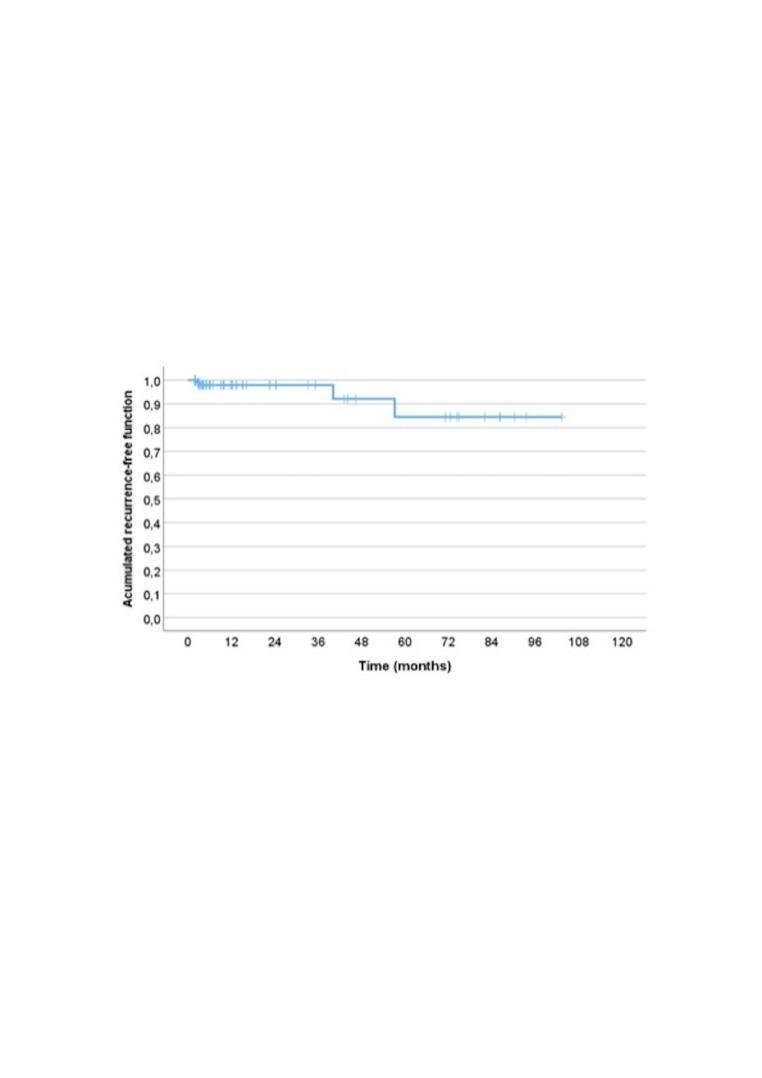
Kaplan-Meier curve showing the recurrence-free period time.

The recurrence-free interval time (RFIT), evaluated using Kaplan-Meier analysis, was 94.023±4.939 months, with a 95% confidence interval of 84.34 to 103.70 months (Figure 4). At 12 months, 97.9% of patients were recurrence-free, and at 5 years, the recurrence-free rate remained high, at 84.4%, indicating excellent long-term outcomes for most cases.

4

No statistically significant associations were found between recurrence-free survival and variables except for the presence of scar (p=0.015) and the type of handpiece (only considering Nd:YAG laser cases) (p=0.04) (Supplementary 1: http://www.medicina.oral.com/carpeta/suppl1_27681). Cases with presence of scar were associated with a lower recurrence-free status comparing with cases without scar. In cases using Nd:YAG lasers, cases using R30 presented a lower recurrence-free status comparing when using 320µm fiber.

To further investigate the relationship between these variables and recurrence, we conducted a multivariate analysis including the significant variables revealed by univariate analysis. In this model, the presence of scar tissue and type of handpiece lost its statistical significance (p=0.498 and p=0.959, respectively).

## Discussion

Oral vascular anomalies, while often benign, can significantly affect patient's comfort, aesthetics, and quality of life, especially when they are visible or located in functional areas such as the lips or tongue. These factors frequently motivate clinical intervention, in particular, with less invasive options compared to classical excision (12 , 13 , 17). In the view of this, we assessed the outcomes of the laser treatment of oral vascular anomalies, with interstitial photocoagulation (IPC) using Nd:YAG, and diode lasers and excision mainly with CO2 lasers. We observed that both surgical excision and interstitial photocoagulation (IPC) techniques were employed without significative complications and appeared effective in eliminating vascular anomalies in our sample composition.

In our study, most cases were diagnosed as venous lakes (75.5%), with a marked predilection for the lower lip, consistent with the literature (13 , 17). The cohort had a slight female predominance, with most patients aged 60 years. No significant association was observed between gender, age, or lesion size and the principal outcome variables such as recurrence or scar formation.

Three laser types were used, with the Nd:YAG laser being the most prevalent (72.4%), followed by CO2 (17.3%) and diode laser (10.2%). The Nd:YAG laser is well-suited for the treatment of oral vascular anomalies due to its high absorption by hemoglobin and its deep tissue penetration (12 , 16 , 19). The diode laser also performed well, offering deep thermal coagulation with selective haemoglobin absorption, particularly suitable for low-flow lesions (13 , 18 , 21 - 23). The CO2 laser, although less frequently used, showed good results for its precision and surface-level haemostasis, taking advantage of the high absorption of water, ideal for excision of small and well-defined lesions. However, its coagulative depth is more limited, making it less effective for deep vascular structures (19 , 24 , 25).

On the other hand, IPC was the most frequently applied technique (80.6%). The type of intervention did not significantly influence recurrence or scarring, highlighting that lesion characteristics may play a more critical role than device or technique selection alone. However, surgical excision performed was significantly associated with more postoperative pain and prolonged healing when compared to IPC (p&lt;0.001 and p=0.026, respectively) in univariate analysis. Also, within the IPC group, the type of handpiece also appeared to influence outcomes: the Fiber HP was associated with longer healing times compared to the R30 HP, but demonstrated a lower recurrence rate, although without significant in multivariate analysis. We may hypothesise that with less energy accumulated and less number of repetitions using R30 a less cicatrisation time and complications will be present but in some cases the energy delivered could not be sufficient in one session comparing to the use of a fiber with a defocused and higher energy application (Table 2). Nevertheless, in the literature IPC has been shown to be a safe and useful procedure as we observed in our sample nevertheless the instruments used (9 , 12 , 13 , 16 , 18 , 25 - 27). Larger and more deep lesions could benefit from endoluminal laser interventions (26 , 28).

Table 2

Lesions size significantly impacted postoperative outcomes, particularly pain on the first postoperative day and healing time, with larger lesions associated with greater discomfort and delayed recovery. However, it was not associated with long-term recurrence, affirming the stability of laser-based interventions.

We observed also a relation between postoperative pain, and the likelihood of scar formation. Scarring, in turn, was linked to recurrence both in univariate and multivariate analyses. Kaplan-Meier analysis confirmed these findings, with patients exhibiting scars showing significantly shorter recurrence-free survival. This suggests that scar formation may be a surrogate marker of healing complications or lesion recurrence. This could be explained by bigger and more complex vascular lesions that result not only in a higher risk of scar presence but also of recurrence.

The recurrence rate was very small in the sample composition. The Kaplan-Meier analysis showed an overall recurrence-free survival rate of 97.5% at 12 months and 88% at 5 years. This aligns with the findings of Bekhor et al., (29) who reported a 94% complete clearance rate after a single Nd:YAG session in 34 cases of venous lakes, without postoperative complications or recurrences. Similarly, Trafalski and Jurczyszyn highlighted the effectiveness of diode and Nd:YAG lasers, identifying them as the most reliable methods for venous lake treatment in the oral mucosa (21). By contrast, Nammour et al. (25) observed higher recurrence rates in their cohort, with 11% recurrence using diode laser and 8% using Nd:YAG laser. Their use of Er,Cr:YSGG and CO2 lasers yielded no recurrence at 12 months, suggesting that laser type and energy delivery method may significantly influence long-term outcomes. Patients should be followed up to confirm effective treatment of these anomalies. A longer follow-up is particularly advisable for lip anomalies, especially venous lakes, as they may share the same etiologic factor as actinic keratosis-an important oral potentially malignant disorder (OPMD) that can coexist with vascular anomalies of the lip (30).

The high satisfaction rates and rapid healing times are also consistent with those reported in the literature (13 , 18 , 19 , 22 , 25 , 26 , 29). These results support the clinical performance of interstitial photocoagulation (IPC), which was predominantly employed in our sample (80.6%) and reinforces the minimally invasive nature of these procedures and their suitability for outpatient management specially for lesions with size &lt;0.6cm. However, variations in laser protocols, lesion types, and follow-up periods make direct comparison difficult and reinforce the need for standardized treatment guidelines.

However, several limitations must be acknowledged. Firstly, the retrospective design introduces inherent biases-such as inconsistent documentation and variability in operator decision-making. The choice of laser or technique was not standardized and may reflect individual authors clinician preference or equipment availability in our Laser Unit. Secondly, only 15.3% of lesions underwent histopathological confirmation, only available for cases submitted to excision. Although clinical diagnosis of venous lakes is typically reliable, especially for lower lip lesions, the absence of routine histology is always a limitation and should be considered before the option of selection the type of intervention. Moreover, patient-related variables such as alcohol intake, smoking status, systemic disease, or oral hygiene were not consistently recorded, limiting analysis of external influences. Finally, although statistical analyses were performed rigorously, the small number of recurrence events (n=4) potentially limits the power of inferential statistics. Although a significant association between scarring and recurrence was observed, this result should be interpreted with caution due to the small sample size. This highlight the need for prospective studies with standardized methodologies and randomized groups of intervention.

## Conclusions

In conclusion, laser therapy in particular, IPC, proves to be a safe, efficient, and minimally invasive option for the treatment of small oral vascular anomalies. Its favourable clinical profile, including the high patient satisfaction, minimal postoperative pain, rapid healing in most cases, minimal postoperative complications and a low recurrence rate supports its integration into routine practice. Future prospective studies with standardized classification systems, histological correlation, and broader data collection are warranted to further define optimal treatment strategies and long-term outcomes.

## Figures and Tables

**Table 1:** Table Variables of the lesions included in the study.

Variable	Groups	n	(%)
Gender	MaleFemale	6150	55.045.0
Age score(year-old)	0-59>=60	5754	51.448.6
Location	LipBuccal mucosaeTongueMaxillary Vestibule mucosaeGumPalate	81129531	73.010.88.14.52.70.9
Size score (cm)	<0.6>=0.6	5457	48.651.4
Diagnosis	Venous lakePyogenic granulomaVascular malformationSpider AngiomaAngiofibromaVascular anomalyRuby Angioma	771210191	69.49.91.89.00.98.10.9
Type of surgery	ExcisionIPC	3279	28.871.2
Type of Laser	CO2 laserNd:YAG laserDiode laser	227811	19.870.39.9
Handpiece (for IPC only)	Mirror HPFiber HPR30 HP	226524	19.858.621.6
Pain 1º day VAS (0-10)	012345>more	822511650	73.91.84.59.95.44.50.0
Pain 3º day VAS (0-10)	012345>more	91388010	82.02.77.27.20.00.90.0
Pain 7º day VAS (0-10)	012345>more	108300000	97.32.70.00.00.00.00.0
Need for medication	NoYes	8823	79.320.7
Cicatrisation time (days)	0-78-1415-21>21	913872	8.111.778.41.8
Cicatrisation time Score (weeks)	<21>=21	2388	20.779.3
Postoperative Complications	NoYes	9912	89.210.8
Scar score	012345	10144200	91.03.63.61.80.00.0
Scar presence	NoYes	10110	91.09.0
Satisfaction score	12345	1075845	0.90.06.352.340.5
Recurrence	NoYes	1074	96.43.6

IPC: Induced photocoagulation. VAS: Visual analogue scale. HP: Handpiece.

**Table 2:** Table Main advantages and disadvantages of the instruments used in the present work.

Laser	Advantages	Disadvantages
CO2 Laser	- Precise and fast incision- Indicated for excision and ablation procedures, superficial and soft tissue lesions- Excellent haemostasis, creating a bloodless surgical field- Allows a non-contact and delicate work- When performing excision, a specimen is available for histological evaluation.	- Nonselective for haemoglobin, therefore not indicated to IPC in vascular anomalies.- If not controlled, can produce a high thermal effect on tissues.- In comparison with IPC (diode or Nd:YAG lasers) could be related with more pain and more time of tissue healing- Expensive to buy, to maintain, and not easily portable.
­Diode Laser (980 nm)	- Portable, affordable and a more accessible instrument;- Good haemostasis and dry surgical field capacity specially for vascular anomalies related with having haemoglobin as chromophore;- When used in a IPC in comparison with an excision (CO2 lasers) is related with less feeling of pain and faster healing time	- Less precise and slower cut/ablation when comparing with CO2 cut- High thermal effects depending on their use;- If IPC, don´t give a histological specimen
Nd:YAG Laser	- Deep tissue penetration- Good haemostasis and dry surgical field capacity specially for vascular anomalies related with having haemoglobin as chromophore- When used in a IPC in comparison with an excision (CO2 lasers) is related with less feeling of pain and faster healing time.	- Less precise and slower cut/ablation when comparing with CO2 cut- High thermal effects depending on their use- Expensive to buy, and not easily portable.- If IPC, don´t give a histological specimen
IPC with fiber	- Equipment more affordable- Good results in only one session protocol- Easily protocol and immediate visible effect	- Potential high level of tissue accumulated energy and high thermal effects depending on their use.- Potential more healing time
IPC with 4-mm spot (R30)	- Faster procedure- Faster healing time- Reduced time of energy exposure	- Could need for more sessions or higher energy delivery to avoid recurrence- Additional cost to standard laser equipment

IPC: Induced photocoagulation.

## Data Availability

Declared none.
